# The Guardian Node Slow DoS Detection Model for Real-Time Application in IoT Networks

**DOI:** 10.3390/s24175581

**Published:** 2024-08-28

**Authors:** Andy Reed, Laurence Dooley, Soraya Kouadri Mostefaoui

**Affiliations:** School of Computing and Communications, The Open University, Walton Hall, Milton Keynes MK7 6AA, UK; laurence.dooley@open.ac.uk (L.D.); soraya.kouadri@open.ac.uk (S.K.M.)

**Keywords:** slow DoS, internet of things, slow read, slow post, slow HTTP get, guardian node

## Abstract

The pernicious impact of malicious Slow DoS (Denial of Service) attacks on the application layer and web-based Open Systems Interconnection model services like *Hypertext Transfer Protocol* (HTTP) has given impetus to a range of novel detection strategies, many of which use *machine learning* (ML) for computationally intensive full packet capture and post-event processing. In contrast, existing detection mechanisms, such as those found in various approaches including ML, artificial intelligence, and neural networks neither facilitate real-time detection nor consider the computational overhead within resource-constrained *Internet of Things* (IoT) networks. Slow DoS attacks are notoriously difficult to reliably identify, as they masquerade as legitimate application layer traffic, often resembling nodes with slow or intermittent connectivity. This means they often evade detection mechanisms because they appear as genuine node activity, which increases the likelihood of mistakenly being granted access by intrusion-detection systems. The original contribution of this paper is an innovative *Guardian Node* (GN) Slow DoS detection model, which analyses the two key network attributes of packet length and packet delta time in real time within a live IoT network. By designing the GN to operate within a narrow window of packet length and delta time values, accurate detection of all three main Slow DoS variants is achieved, even under the stealthiest malicious attack conditions. A unique feature of the GN model is its ability to reliably discriminate Slow DoS attack traffic from both genuine and slow nodes experiencing high latency or poor connectivity. A rigorous critical evaluation has consistently validated high, real-time detection accuracies of more than 98% for the GN model across a range of demanding traffic profiles. This performance is analogous to existing ML approaches, whilst being significantly more resource efficient, with computational and storage overheads being over 96% lower than full packet capture techniques, so it represents a very attractive alternative for deployment in resource-scarce IoT environments.

## 1. Introduction

The increasingly pervasive nature of the *Internet of Things* (IoT) paradigm in information and communication technologies means web-based IoT nodes, networks, and systems are penetrating evermore diverse application areas ranging from industrial, agricultural, and manufacturing to e-health care and home automation. The drive, however, to mass produce high volumes of inexpensive IoT devices inevitably means that security functionality has become compromised. In many cases, this inadequacy extends to the critical network ingress point, with IoT gateway nodes often having minimal security provision for intrusion detection [1]. There are currently three prevailing IoT communication protocols, namely *Hypertext Transfer Protocol* (HTTP), *Constrained Application Protocol* (CoAP), and *Message Queuing Telemetry Transport* (MQTT), and while the latter two are designed to be more resource efficient, the dominance of HTTP in IoT vendor markets [2] demonstrates the critical importance of being able to reliably secure HTTP-enabled IoT nodes.

IoT security vulnerabilities pose serious technical challenges, none more stark than in e-health, where disruption or a DoS preventing users or systems from accessing critical medical data can compromise patient well-being. Due to the growth of IoT applications in this domain, DoS attacks present a major threat, as highlighted in [3]. The corollary is that this has given significant impetus to developing effective mitigation strategies to prevent malicious IoT node activity. *Denial of Service* (DoS) attacks are a major network security threat in terms of their impact and detection difficulty [4]. They are particularly acute in IoT scenarios, where devices are usually resource scarce in their computational power, battery life, and storage capacity [5]. These serve to limit the applicability and deployment of existing DoS detection systems to IoT networks, and thereby render these highly susceptible to such malicious attacks [6]. Low-bandwidth DoS attacks, known as Slow DoS, have received much attention due to their operational simplicity [7] and accessibility of tools to launch them. A notable risk of Slow DoS is their propensity to replicate benign HTTP requests from *legitimate nodes* (LNs), with their slowness often resembling a genuine node facing intermittent or poor connectivity [8]. Slow DoS attacks thus present a significant challenge by exhibiting stealthier traffic profiles, which makes them hard to detect using traditional methods compared to high-volume DoS or *Distributed DoS* (DDoS).

The three main Slow DoS variants that target HTTP applications are *Slow Get* (SG), *Slow Read* (SR), and *Slow Post* (SP) [8], with each, respectively, concentrating on a unique functional HTTP parameter to initiate malevolent behaviour. The corollary is that each variant displays different network characteristics, so it is very challenging to design a generic Slow DoS detection solution [9], with the problem intensified in resource-constrained IoT environments. The anatomy of a Slow DoS attack is inexorably linked to the connection-orientated nature of *Transmission Control Protocol* (TCP), where each client-to-server connection is held open to allow the upper-layer protocol to function. Once a TCP connection is made, all client and server requests and responses are initiated to set key parameters governing data exchange, such as window size and flow control. TCP ensures the web server waits until the upper layer application, such as HTTP, either completes the required tasks or until a local timeout expires and the connection is closed. It is this connection-orientated design that Slow DoS attacks exploit, emphasising why such malicious activity on web-based IoT nodes is a major security vulnerability, a threat compounded by the challenge of reliably distinguishing them from legitimate HTTP requests [10]. Slow DoS attacks are characterised by nominal bandwidth usage as payloads are often trivial, though their impact in rapidly consuming limited resources is significant. They also alias as genuine TCP and HTTP traffic, which is exacerbated whenever LNs have erratic or poor connectivity. Slow DoS substantially reduces the operational functionality of low resource IoT nodes, even in more robust Apache HTTP installations [11], by sending HTTP exchange requests and responding very slowly so available web server resources are quickly expended.

Various intrusion-detection mechanisms have been proposed to reliably identify Slow DoS attacks, with the most common predicated on network traffic anomalies [9], involving bi-directional packet inspection. Other anomaly detection approaches use *Machine Learning* (ML) [12], *Artificial Intelligence* (AI) [13], and most recently, *eXplainable AI* (XAI) [14] techniques. A major drawback of these ML approaches is the computational resourcesincurred to create the high volumes of network activity necessary to build a large dataset of usable attributes reflecting different performance metrics and traffic conditions. There is also the added cost of continually retraining ML systems [1], while [15] concluded these drawbacks were collectively undermined using ML as a real-time detection mechanism for resource-constrained IoT nodes.

Many ML-based intrusion-detection approaches are also strongly influenced by the performance variations caused by the diverse datasets they are tested upon [16]. Overfitting is a recurring problem, while the inability of trained models to recognise and adapt to varying *malicious node* (MN) attack parameters leads to the *concept drift* phenomenon [17], which can seriously compromise a detection model’s accuracy, an issue especially challenging in IoT networks.

The original contribution delineated in this paper is a dedicated, computationally lightweight, resource-efficient *Guardian Node* (GN) which forms the basis of a generalised Slow DoS detection model for IoT environments. The IoT gateway node or *Border Router* (BR), which is responsible for forwarding all inbound and outbound network packets, delegates to the GN, the critical role of accurate, resource-efficient detection of all three key Slow DoS variants. A fundamental resource benefit of the GN model is the requirement to inspect only inbound packets of a specified length, while the efficiency stems from the judicious real-time, two-stage design, which inspects just two key attributes to identify malicious SG, SR, and SP attacks without erroneously denying network access to either LNs or *Slow Nodes* (SNs), having high latency or poor connectivity. In essence, the GN is a resource-efficient alternative to the computationally expensive back end or remote servers prevalent in existing Slow DoS detection solutions and thereby affords a significant security enhancement for energy-scarce IoT networks.

The quantitative performance of the GN Slow DoS detection model has been critically evaluated with results consistently demonstrating high, real-time detection accuracy comparable with existing computationally intensive ML solutions, whilst incurring significantly lower resources. Key parameters can be fine tuned to detect Slow DoS attacks across a raft of stochastic IoT traffic profiles, while crucially, it reliably identifies the stealthiest malicious Slow DoS activity which mimics the behaviour of a genuine SN with poor connections. This set uniquely distinguishes the GN model from existing anomaly detection methods and ML classifiers as a security tool tailored to IoT environments, where low resource expenditure is imperative.

The rest of this paper is organised as follows: Section 2 reviews the current state-of-the-art in Slow DoS detection techniques, while Section 3 details the new GN Slow DoS detection model. Section 4 describes the experimental IoT test network platform used, with Section 5 presenting a critical evaluation of the performance of the GN detection model. Section 6 presents a comparative resources analysis with existing ML-based solutions, with Section 7 providing some concluding remarks and future work directions.

## 2. Related Work

Many IoT devices are inherently resource constrained, so security functionality tends to be a subordinate requirement with intrusion detection often left to the IoT gateway node of a network. IoT gateways, however, are often inadequately equipped in their security capability [18], with scarce computational resources to support current intensive intrusion-detection algorithms involving AI, ML [12], and deep learning [19]. Whilst the ensuing detection rates are encouraging, these approaches all mandate off-line, bi-directional, post-event analysis, and as [20] highlighted, are incompatible with IoT scenarios due to the high resource overheads. A lightweight neural network model for real-time detection suitable for IoT gateway nodes was proposed in [1]. Using a combination of high-specification computers and Raspberry Pi devices, an empirical evaluation demonstrating high detection accuracy was achieved for several HTTP attacks, including SG, though the findings concomitantly emphasised the high computational costs incurred by running the model in a live IoT network. A key consideration here is the resource requirements to conduct *Full Packet Capture* (FPC) involving both inbound and outbound packet processing. FPC is highly resource intensive and common in ML models, as evidenced in [10,21].

Slow DoS detection using ML techniques generally means assembling high-volume datasets of post-event, bi-directional network activity to generate a measurable dataset, such as CICIDS2017 [22], where 80 different attributes are extracted for analysis. These ranged from basic network information like node addressing, protocols, and packet length, to more granular information on network conditions. In such high-volume datasets, cognisance must be made of the corresponding resources incurred in either capturing such a huge amount of network traffic or ingesting and processing existing open-source datasets. A wide-ranging assessment of synthetic datasets was conducted by [23]. This not only stressed their ability to reflect malicious activity in IoT systems but equally highlighted the hiatus in both real IoT node traffic and nuanced attack profiles.

Dataset size is a key factor in ML approaches, where training, testing, and validation times commensurately impact detection rates, and while there are advantages of post-event analysis of large datasets comprising multiple network attributes in terms of detection accuracy, their high storage and computational time requirements, allied with the need to continually retrain the model, inhibits their applicability in resource-constrained IoT environments. Indeed, the resource cost of dataset acquisition and processing in ML solutions is often ignored and viewed purely as a black box [1] with limited insights or understanding as to how the various individual attributes influence a particular model’s detection decisions. In [13], a combined AI anomaly-based Slow DoS detection framework using deep learning techniques and real-time traffic analysis was proposed. This involved several computational layers and the synthesis of a dataset of 57 attributes for analysis, thus expending sizable resources. The drawback from a practical IoT perspective is that this approach imposes a prohibitively high hardware overhead of three extra devices to support the monitoring node, each having 8 GB of storage and 16 core processors.

An alternative to processing high-volume datasets was proposed in [24], where throughput improvements were achieved using just the attributes of packet length and TCP segment size. This had the benefit of lower computational overheads in data aggregation while demonstrating comparable accuracy to ML detection models. Extending this approach was successfully demonstrated in [11], which presented an IoT SG detection framework using the dual network attributes of packet length and delta arrival time. A novel aspect of this solution was the explicit introduction of an SN category to reflect genuine IoT nodes with high latency connections. Results corroborated that the framework consistently differentiated LNs, including SN packets, from MN packets, while upholding detection accuracy levels commensurate with existing ML methods. The framework methodology, however, was specifically tailored to Slowloris (SG) attacks, and while it exhibited the benefits of real-time detection and attribute efficiency, resource expenditure was significant as every inbound packet had to be processed. Furthermore, only one live IoT traffic profile was synthesised in the test dataset, so it did not reflect the typical breadth of stochastic IoT network conditions.

Another restriction of existing SG detection mechanisms like those in [7,11] is their implicit reliance on default MN attack parameters, so identifying either non-default or more stealthy SG attack profiles would symbolise a noteworthy advance in Slow DoS detection practice.

In reviewing the respective traits of the three Slow DoS variant attacks, SG acts by sending a partial HTTP GET request to open a connection; however, the MN deliberately fails to complete the request, with the server keeping the connection open until a timeout is reached [25]. Generating multiple concurrent connection requests inevitably leads to the MN occupying all available server threads. SR exploits the TCP window size attribute, which governs the amount of data transmitted and received [8]. The MN reads server responses slowly while holding the connection open by advertising a trivial window size value, again occupying available threads and degrading server performance. In contrast, SP exploits the content length field of the HTTP header by negotiating large data transfers, but instead, the MN only sends trivial byte values, keeping the connection open long enough to exceed the server time-out [26].

Narrowing this time-out value or limiting connections on a per-node basis have been proposed as mitigation strategies [8], although both approaches can negatively impact IoT SN connectivity because slow legitimate requests are incorrectly timed out. In addition, continued probing of the server can easily flag to an MN that key connection configurations have been changed, allowing Slow DoS attack parameters to be adapted accordingly. Despite the obvious benefits of existing Slow DoS detection proposals, this critical review has contextualised a significant gap in developing a generalised, resource-efficient methodology for IoT scenarios, which not only accurately detects all Slow DoS attack variants in real time, but crucially has the ability to dependably discriminate LNs and SNs from MN activity. This provided the motivation to design a unified GN Slow DoS detection model, which can accurately and efficiently identify the SP, SG, and SR Slow DoS variants within resource-constrained IoT environments. The model innovatively synthesises and extends the limited attribute methodology of [11,24] to realise a detection strategy that can uniquely respond in real time, to stealthy adjustments in both MN attack parameters and dynamic IoT network traffic conditions. The GN detection model design will now be explained in detail.

## 3. The GN Slow DoS Detection Model

To address the critical issues of identifying Slow DoS attacks in resource-constrained IoT networks, this paper presents a GN Slow DoS detection model, which both functions in real time and is resource efficient so it is able to reside on a single node in an IoT network. By analysing in real time the key network attributes of *Packet Length* (*lp*) and *Delta Time* (Δt), the GN model uniquely embraces all three Slow DoS attack variants in a single unified detection methodology, notably extending the detection capability beyond the single-variant, SG attack approach proposed in [11]. The model will be shown to robustly operate even in the most stochastic of IoT traffic environments, where node resources can be severely limited. Furthermore, by narrowing the *lp* threshold range and adaptively tuning the Δt window, processing overheads are substantially lowered, with the corollary being a generalised resource-lightweight Slow DoS detection solution.

**Figure 1:**
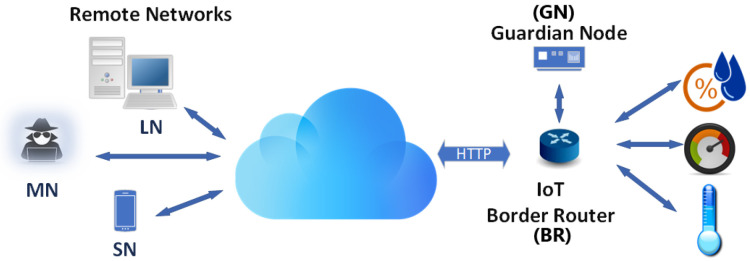
The Guardian Node (GN) network topology.

Figure 1 conceptualises the IoT network topography, with the functional relationship between the BR and GN illustrated. The primary role of the BR is forwarding all inbound packets whenever two key criteria are upheld: (i) when the IP address and TCP port number already exist in the BR forwarding table, and (ii) the sender IP address fails to meet the conditional *Deny* statement in the *Access Control List* (ACL). The GN, which is a lightweight IoT node, only examines inbound packets that fulfil a specified *lp* value and evaluates.

The legitimacy of a packet is determined solely by inspecting the *lp* and Δt attributes.It then updates the ACL of the BR with the outcome to either permit or deny network access. This selective packet inspection is a significantly more efficient methodology than both high-volume dataset-driven approaches and conventional intrusion-detection systems, where every packet is inspected, and often in a bidirectional manner as required in ML, AI, and XAI techniques. The inclusion of a separate GN as an adjunct to the BR delivers several advantages. Aside from being significantly more resource efficient by reducing BR processing overheads, it both mitigates the omnipresent risk of a single point of failure [27] at the IoT gateway, while its out-of-band placement facilitates real-time packet inspection. The compromise, however, is that the GN is a resource imposition on the IoT network, though as Section 6 compellingly validates, the additional cost incurred is minimal, and more than offset by the performance benefits.

The pseudo code for the GN Slow DoS detection model is presented in Algorithm 1. Both *lp* and Δt values are measured at the ingress point of the GN (Lines 4 and 11, respectively), with the initial range of *lp* values analysed for potential indicative anomalous behaviour. *lp* analysis is predicated on the premise that Slow DoS enforces a slow connection by sending either partial or incomplete requests and responses to the Web server [10]. All packets with a *lp* value that fall within a designated *threshold_range* are labelled *candidate MN* for further inspection. The remaining packets are marked as legitimate and granted network access by the BR. To mitigate the possibility of both LNs and SNs being misclassified during *lp* scrutiny, all *candidate MNs* undergo further processing with their contiguous Δt values within a stream being measured. In the context of packet analysis, the stream relates to a collection of packets unique to a single TCP conversation, with each packet belonging to a stream given a numerical value called the *stream index*. This ensures a reliable mechanism for analysing metrics and attributes bound to a TCP single conversation. To identify anomalies in packet transmission times, the respective Δt values are analysed since the intrinsic aim of a Slow DoS attack is to keep the client server connection open and degrade server performance and availability. Δt is formally defined as

(1) $$\Delta t=t_{a_{k-1}}-t_{s_{k}}$$

where $t_{a_{k}}$ and $t_{s_{k}}$ are, respectively, the observed arrival and start times of the *k*th observed packet (Line 11). Packets where Δt exceeds a prescribed threshold $\Delta t_{Th}$ (Line 12) are labelled malicious and denied network access by the BR. Analysing $\Delta t_{Th}$ provides an innovative method of being able to clearly demarcate between LNs, SNs, and MNs, as will be evidenced in greater depth in Section 5.
**Algorithm 1** Guardian Node Inbound Packet Classification 1:**Input:** Inbound LN, SN, MN packets 2:**Initialise:** *lp threshold-range*; $\Delta t_{Th}$; *candidate MN* 3:**Output** Permit LN or SN and deny MN access 4:**Repeat** Calculate *lp* of each packet 5:**if** *lp*∈ {*lp threshold-range*} **then** 6:      label as *candidate MN* 7:**else** 8:      LN or SN and permit access 9:**end if**10:**Until** All packets processed11:**Repeat** Calculate Δt of each *candidate MN* using (1)12:**if** Δt $\geq \Delta t_{Th}$ **then**13:      classify as an MN and do not add to ACL14:**else**15:      classify as LN/SN and add to ACL16:**end if**17:**Until** All *candidate MN* processed

## 4. Live IoT Network and Dataset Synthesis

Section 2 mentioned several publicly available datasets appertaining to IoT and Slow DoS attacks, though none of these take cognisance of SNs encountering high latency or poor network connectivity, nor do they contain MN attack traffic beyond default parameter settings. This latter trait significantly limits current detection strategies for such datasets, especially when the MN parameters are configured to produce highly stealthy attack scenarios. This provided the impetus to construct a new dataset comprising all three Slow DoS attack variants together with genuine SN-generated traffic within a live IoT network.

While data capture on a live IoT *production environment* was considered, it was not a viable option because of the potential for unintentional data protection breaches and regulatory restrictions [14].

The corresponding HTTP IoT dataset is available at [28]. In creating this dataset, live network events were synthesised using low resource nodes, within a multi-routed topology replicating remote connections passing through multiple network devices. The remote nodes (LNs, SNs, and MNs), along with the GN, are represented by Raspberry Pi model 4 boards, each having 4 GB RAM, 16 GB flash, and a 1.2 GHz CPU. In contrast, the resource capacity of the IoT sensor nodes used has only 18 K RAM, 256 KB of flash memory, and a 20 MHz CPU, which significantly limits their ability to process concurrent HTTP requests. The BR is similarly resource constrained with only 128 MB DRAM and 64 MB of flash.

Wireshark, which is a well-known inspection application, is used to filter network packets from each node [20]. The resulting dataset includes the Wireshark trace file (in .PCAPNG format) together with both supplementary information on the attack parameter settings for each Slow DoS variant and the corresponding node IP addresses. The IoT sensor nodes included a DHT22 humidity monitor, connected via a Raspberry Pi model 4 board, a TME-critical temperature probe with a threshold alerting function, and a Papago 2TH Wi-Fi twin thermocouple, humidity, and dew point sensor. All these sensors allowed live data to be read via HTTP GET, while the TME sensor allows live data to be received via HTTP POST in XML format. The Apache service was installed as the web interface, with standard default security settings and operational parameters that support the DHT22 sensor. Both the 2TH and TME sensors were preinstalled with a basic HTTP script as part of the node firmware. The HTTP service of each node provided clients with remote access to the critical data readings produced by the sensors in 1 s intervals. The resulting trace file afforded an effective method of extracting the key network attributes from [20]. The SlowHTTPtest utility was installed on the designated MN and configured to consecutively launch SG and SR attacks. For the SP attacks, Switchblade 4.0 was installed on the MN, as this application offered greater control of how the HTTP data are posted to the server [7], ensuring a successful Slow Post Attack is achieved.

### 4.1. Justification for IoT Environment Software and Analysis Methods

To analyse the GN model performance, the requisite live network attributes with their respective values are extracted from the Wireshark trace file .PCAPNG format and converted into a .csv format. This allows regression testing, modelling, accuracy prediction, and statistical analysis using the *Statistical Product and Service Solutions* (SPSS).

Package which has been widely adopted, such as for intrusion-detection modelling of black hole attacks [29]. To compare the GN model accuracy and related confusion matrix metrics, the Weka application was employed because it is extensively used in ML classification and data mining, with a large suite of library routines for synthesising supervised and unsupervised Slow DoS detection ML models as evidenced in [30,31], and recently detecting DoS in IoT environments using dimensionality reduction techniques [32]. The Weka framework, along with supporting libraries is available at [33], while the complete IoT Slow DoS dataset and full documentation for the GN model can be downloaded from [28].

### 4.2. Scalability Analysis

As discussed in Section 4, the network architecture synthesised for this evaluation of Slow DoS attack scenarios is an environment for capturing and analysing live traffic, with the network topology able to be easily scaled to multiple sub-networks. To scale intrusion detection in IoT environments, node clustering [34] can be employed to reduce or redistribute node resources, though clustering generally requires centralised management to maintain cluster node continuity. The IoT network topology design adopted is a more lightweight approach to support scalability using load balancing [35], with the BR configured to balance the traffic load between multiple sub-networks, with each sub-net having a dedicated GN for MN detection.

### 4.3. Traffic Profiles

To construct a more robust test environment, three pragmatic *traffic profiles* (TPs) were synthesised for each Slow DoS variant. The high-volume attack (TP1) is the least stealthy profile and the default for the SlowHTTPTest application. To evade Slow DoS detection, the TP2 profile reduces the number of attack connections to 1000 so this is a stealthier MN attack, while the final profile TP3 has just 500 connections generated by the MN, and thus is the stealthiest attack profile. To further mask the detection of a Slow DoS attack, the MN was configured to launch various attack parameters measured in *Requests Per Connection* (RPC). These parameters, such as the request intervals, were variable, though once a Slow DoS attack was launched, it was assumed these attack parameters were fixed.

Table 1 summarises the default parameters for each Slow DoS variant in terms of the numbers of *TCP connection requests* (cnx) and HTTP *requests per connection* (rpc). In order to imitate a more stealthy attack, alternative connection parameters for each Slow DoS variant were generated. Table 1 highlights the reduced connection parameters used mirroring the approach in [13], where the attack connections for each variant’s connection rate are reduced by 50% from high to low volume. In [13], three distinct DoS attack datasets were created for high, medium, and low connection rates. The three unique TPs generated datasets for the GN model analysis adopted a similar approach, though in [13], the low connection dataset failed to launch a successful DoS attack, whereas TP3, which is the most stealthy attack profile, was able to launch a complete DoS during the attack period.

At this point, it was important to attempt to maintain an unbiased class balance for the datasets constituent to each TP; this was necessary to provide an equitable comparative analysis.

### 4.4. Class Frequency Analysis

It can be seen in Table 2 that TP1 produces a class balance of 54.3% for the three Slow DoS variants combined into the MN class, along with SNs and LNs combined, representing 45.7%. Whilst this gives a pragmatic class balance, this profile is indicative of a non-stealthy attack scenario.

The traffic generated by all nodes for TP2 in Table 3 displays a significantly more unbalanced frequency of node activity compared with TP1, highlighting a disproportionate bias to the majority class of the MN. The importance of maintaining an equitable class balance for dataset analysis in DoS attack detection is discussed in [36], and is implemented as part of a Slow DoS detection model in [37].

As seen in Table 4, TP3, the highly stealthy profile, provided a split of 40.7% for LNs and SNs combined against 59.4% for MNs, as the three combined Slow DoS variants. This profile affords a comparable class balance between the LN and MN classes, whilst providing a viable subset of traffic attributes under highly stealthy attack conditions, of which analysis could be conducted.

### 4.5. Delta Time Comparison for Each Traffic Profile

The comparative mean $\Delta_{t}$ values in ms, presented in Table 5, illustrate how revising the Slow DoS parameters impacts the appearance of each variant. It is apparent that by reducing the Slow DoS connection parameters, the corresponding mean $\Delta_{t}$ between packets also decreases. Applying linear regression confirmed the relationship between reducing attack connections and lower mean $\Delta_{t}$ per MN. The regression provided a strong correlation between the variables with $R^{2}=88\%$, which corroborates the hypothesis that further reductions in attack parameters lead to a mean $\Delta_{t}$, converging with that of an SN. By comparing the respective SN and SP values for TP3, there is cogent evidence that further convergence will eventually lead to indistinguishable $\Delta t_{\mu }$ values. Table 5 illustrates how Slow DoS attacks can evade detection and as highlighted in Section 2 and [8], demonstrates why aggressively configuring server wait and timeout parameters without cognisance of constituent node connectivity constraints can easily lead to genuine SNs being erroneously classified as MN.

To synthesise realistic SN activity, variable latencies were introduced into the network traffic by injecting packets with random delays of between 1500 and 3000 ms. The inclusion of SN-generated network traffic is an important aspect in evaluating the harmful impact of Slow DoS activity within IoT environments, where constituent node resources and transmission range limitations are far more acute. To generate large volumes of LN and SN web traffic, it was necessary to create virtual clients using the JMeter application, which is a web server performance-monitoring tool [38]. The LN and SN connections were also modelled on the three TPs to observe variations in attribute values under different network loads, as measured in *page requests per sec* (PRS). Each TP was set to run for a duration of 240 s, with the high-volume TP3 generating 10 PRS, and TP2 and TP1 generating 5 and 1 PRS, respectively. Table 5 also shows that as network load increases, SN experiences higher degrees of latency.

During the Slow DoS attack testing phase, a remote client attempted to read sensor data from each IoT node at 1 s intervals. Testing revealed that even for the stealthiest TP3 profile, during any of the three Slow DoS variant attacks on the sensor node, the remote client was unable to read data between 49.8 and 59.7 s. This cogently demonstrates just how susceptible IoT sensor nodes are to malicious Slow DoS activity, which in a mission critical application where timely access to sensor readings is imperative, can lead to a calamitous outcome. The next section presents a detailed critical performance evaluation of the GN Slow DoS detection model.

## 5. Experimental Results Analysis

To experimentally test and evaluate the performance of the GN Slow DoS detection model, the following attributes were extracted from the new HTTP IoT dataset compiled in Section 4. The packet length *lp* is a network datagram (in bytes) which is the cumulative value of all headers, options, flags, and application data. In contrast, the *TCP segment length (ls)* refers only to the TCP application data, or TCP payload. As highlighted in Section 2, the length of an incoming packet has previously been used for anomaly detection [11], where a known attack exhibits particular characteristics, like the size of the MN payload. By analysing the *lp* values for the various Slow DoS variants, an MN profile can be distilled and, respectively, compared with those corresponding to an LN and SN.

*lp* analysis requires a more in-depth inspection to evaluate the byte-wise construction of packets generated by any node in the network. For each packet counted, *lp* is calculated by summing the number of bytes (*n*) that, respectively, represents the byte count of the *i*th TCP field, including *header* ($h_{i}$), *flag* ($f_{i}$), *option* ($o_{i}$), and *segment length* ($ls_{i}$), as in Equation (2). Observing the *lp* constituents in such granularity affords an accurate means of identifying anomalous values.

(2) $$\mathrm{lp}=\sum_{i=1}^{n}\left(h_{i}+f_{i}+o_{i}+ls_{i}\right)\;\;\mathrm{bytes}$$

By focusing on network events generated by each node type, it was found that all packet lengths exist within a broad range, 60≤lp≤1540. Further investigation revealed that inbound packets in the range of {60, 74} bytes had the highest occurrence, identifying them as candidates for closer inspection. The analysis also showed that *lp* = 66 and *lp* = 74 bytes constituted the highest percentage of packets, respectively, generated by SR, SG, and SP attacks, as confirmed in Table 6, which also includes the corresponding comparative values for both LNs and SNs.

Interestingly, all packets where *lp* ∈ {60, 74} have a *ls* = 0, indicating there is no payload data.

The essence of any Slow DoS attack is manipulating attributes like the TCP window size, with the explicit intent of slowing client–server communications [39]. Window size specifies the amount of data a client can receive, so there is a correlation between the advertised window size and the corresponding *ls* value. Typically, an SR attack is designed to advertise an abnormally narrow window size to slow transmission, and as such, the presence of a disproportionately high number of *ls* = 0 packets is a useful indicator of a potential SR attack. The final *ls* = 0 column in Table 6 affirms this judgement with a notable increase apparent for SR, though solely relying on a *ls* = 0 criterion as the discriminator is more problematic for both the SG and SP variants. This explains the design rationale underpinning the GN model of only considering *lp* within a narrow range *lp* ∈ {60, 74}, where *ls* = 0.

To validate the hypothesis that Slow DoS exhibits a higher proportion of packets where *ls* = 0, an evaluation was performed on the CIC-IDS2017, intrusion-detection dataset [40], with results proving convincingly that for both constituent Slow DoS dataset variants, namely Slow HTTP Test and Slowloris (SG), a higher percentage of packets where *ls* = 0 was discerned for *lp* ∈ {60, 74}.

A key observation from this analysis is that where ls>0, the mean *lp* value is 166 bytes, so inspecting only *lp* ∈ *60, 74* improves the processing efficiency of the GN model as there is no requirement to extract the TCP payload attribute from such packets (*pk*) in the trace file ($\hat{x}$). As such, inspecting only packets in the trace file satisfies the following condition in Equation (3), requiring less processing than inspecting packets where ls>0.

(3) $$\forall pk\in \hat{x}\cap pk:60\leq lp_{pk}\leq 74,ls=0$$

By eliminating unnecessary attribute extraction where ls>0 of inbound packets, the GN model processes ≈ 96% fewer bytes per sec, which significantly reduces the processing overhead performance of the Slow DoS detection model.

### 5.1. Analysing and Labelling Candidate MN

Results confirm that all packets where lp>74 bytes contain some data, so the ls>0 condition precludes all packets where lp>74 from further scrutiny. Table 6 also highlighted that the highest percentage of MN packets resides in 66≤lp≤74, which implies a narrow window of interest, where *lp*∈ {66, 74} can be pragmatically applied to identify and label these packets as *candidate MN* for further examination (Lines 5 and 6 in Algorithm 1).

The results in Table 6 for *lp* = 66 bytes show that both SNs and LNs constitute just 0.6% of all packets of this length. This implies that reliance solely on *lp* analysis can lead to potential misclassifications, albeit nominal, which was the rationale for introducing the *delta time* Δt attribute analysis into the GN model. This second step only inspects *candidate MN* packets to identify transmissions that exhibit a higher-than-expected delay. By attempting to distinguish between genuine SNs and MNs, this both enhances the robustness and accuracy of the MN detection process.

As described in Section 2 and Equation (1), Δt is the time recorded between packets in a contiguous stream during an active client–server communication, which is often used in traffic analysis to indicate packet delays within a given TCP stream.

In this second step, the overall delay (*D*) of packets is measured, as in Equation (4), where the *i*th and *j*th packets are in a contiguous stream.

(4) $$\begin{array}{c} D=\{\Delta t_{ij}:i,j\in \left\{1,2,\ldots ,n\right\},i<j\} \end{array}$$

The GN model calculates the average delay $\Delta t_{\mu }$ between all the *candidate MN* packets as

(5) $$\Delta t_{\mu }=\frac{\sum_{k-1}^{pk}{d_{k}}_{+1}-d_{k}}{pk-1}$$

where $d_{k}$ is the *k*th packet delay time and pk the total number of packets received/observed within the contiguous stream.

To demonstrate the effectiveness of the GN model in exploiting Δt analysis to reliably differentiate between LN, SN, and MN packets, the following evaluation will focus on the stealthiest and most challenging traffic profile TP3 in the live IoT network (Section 4), where without loss of generality, the MN is assumed to be an SP Slow DoS attack.

### 5.2. Calculating the Delta Time Threshold

The $\Delta t_{\mu }$ value (in ms) is calculated for each node, with the particular focus on SNs as this has the greatest danger of misclassification as an SP node. If $t_{k}$ is the arrival time of the *k*th packet and *N* is the total number of packets, then $\Delta t_{\mu }$ for an SN is given by

(6) $$\begin{array}{c} SN\Delta t_{\mu }=\frac{1}{N-1}\sum_{k=2}^{N}\left(t_{k}-t_{k-1}\right) \end{array}$$

**Figure 2:**
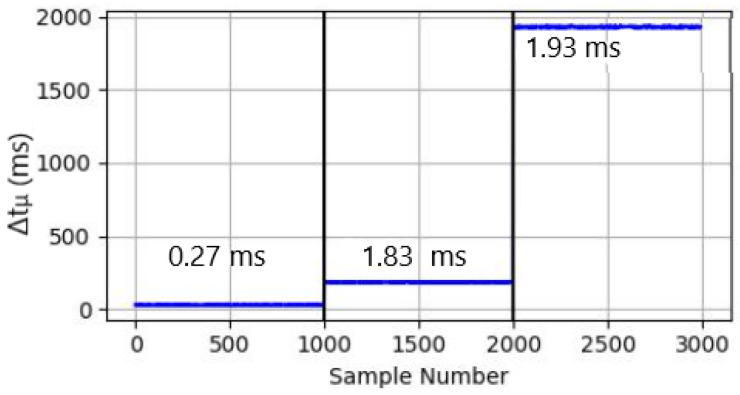
Respective TP3 $\Delta t_{\mu }$ values for LN (left), SN (centre), SP (right).

The maximum *SN*$\Delta t_{\mu }$ and minimum SP $\Delta t_{\mu }$ for TP3 is plotted in Figure 2. The difference between the maximum and minimum values of SNs and SP, respectively, define the *range*(r) such that $\Delta t_{r}=SP\Delta t_{\mu }^{min}-SN\Delta t_{\mu }^{max}$

The results in Figure 2 and Table 5 for the stealthiest MN attack profile TP3 cogently demonstrate the ability of the GN model to reliably distinguish SNs from all *candidate MN*, with, for example, the slowest genuine SN still able to be differentiated from the SP, with a $\Delta t_{\mu }$ margin of 0.1 ms. However, as evidenced in both Section 4 and Table 5, by modifying the Slow DoS attack parameters, the SP $\Delta t_{\mu }$ can synthesise an even stealthier attack, which, when allied with an increased *D* value in Equation (4), can lead to a scenario where *r* narrows to the degree that the SN$\Delta t_{\mu }$ tends to converge towards the minimum SP $\Delta t_{\mu }$, with the corollary being an increased likelihood of misclassification and higher FPR. It can be conversely argued, however, that under more severe network conditions than TP3, while further convergence between SNs and SPs could impact detection accuracy, concomitantly, cognisance should be made of both the maximum SN latency permissible before a server drops its session, and the intrinsic capacity of the SP node to still effect a Slow DoS attack in such challenging circumstances.

Employing the findings distilled in Table 5 and Figure 2 means that the $\Delta t_{Th}$ in Algorithm 1 can be fine tuned and pragmatically set for all *candidate MN* packets to enable MNs, SNs, and LNs to be consistently labelled (Lines 12 to 15 in Algorithm 1).

Controlling $\Delta t_{Th}$ in the GN model thus affords an accurate discriminator of both LN and SN packets, with only a negligibly small probability of SN packets being misclassified as an MN, under all three Slow DoS attack scenarios, across the gamut of traffic profiles. This demonstrably confirms that by judiciously narrowing and refining key parameter values and ranges for the two network attributes analysed by the GN model, all three variants can be reliably identified in real time at the ingress point of an IoT network. This is advantageous, as it removes any necessity for recalculating and retraining times. The next section conclusively demonstrates the suitability of the GN model for IoT environments with a holistic performance evaluation.

## 6. Performance Analysis of the GN Model

By virtue of only having to inspect packets where *ls* = 0, considerable savings in the number of processed packets is achieved, reducing the overall CPU load compared to analysing FPC and inbound packet capture. This lower CPU load contrasts starkly with ML approaches [1], which, for example, generate a 36% CPU load and utilise 114.5 MB of memory. This section quantitatively analyses the accuracy and resource expenditure performance of the GN model. Given the IoT focus, resource efficiency is the paramount requirement, so a critical comparative evaluation of the computational and storage overheads incurred will be examined.

### 6.1. Accuracy

To evaluate the detection accuracy of the GN model, the following six established ML classifiers were utilised as comparators, namely *Bayesian Network*, (BayesNet) [41], *RepTree*, (Reduced Error Pruning) [42], J48 [31], Random Forest [32], *Repeated Incremental Learner* (JRip) [42], and *K-Nearest Neighbour* (KNN) [43]. These ML classifiers were specifically chosen because of their relatively low computational resource requirements, efficiency, and speed, ensuring an equitable comparative analysis with the GN model. Each classifier was evaluated using the Weka data mining and ML application. The test scenario used the most challenging TP3 dataset, with a 70–30% split for training and testing, respectively, and each ML model being trained and tested using only the lp and $\Delta_{t}$ attributes. A pre-processing stage was applied to remove all node IDs after classification. Prior to evaluating the classifiers, the choice of the two key attributes was assessed and validated using the information gain attribute evaluation model. This feature ranking approach to ordering attributes or variables by value is commonly used in ML [44].

**Figure 3:**
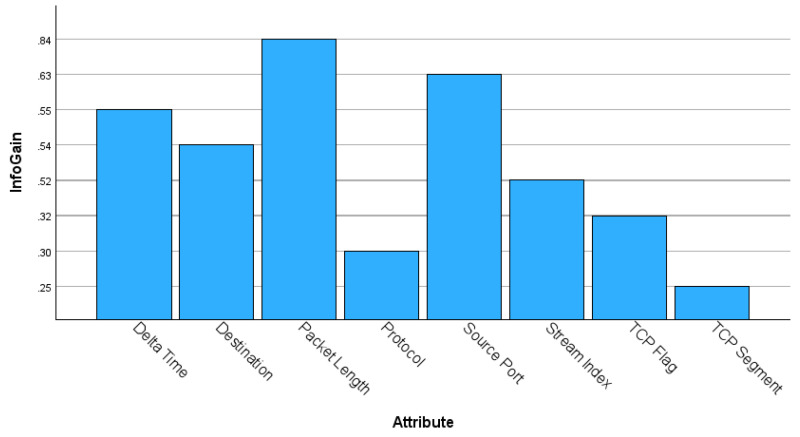
Information gain values for eight common attributes.

The inclusion of an information gain ranking process for eight common attributes afforded a valuable insight into the importance of the *lp* and $\Delta_{t}$ attributes. Figure 3 displays the ranking for each attribute with *lp* ranked highest at 0.844 and $\Delta_{t}$ ranked third at 0.549. It should be stressed that while the source port is a commonly selected feature, as seen in [45], and occurs in many DoS datasets including Bot-IoT [23], this attribute can be problematic, and may cause misleading results or classification errors due to it being tightly associated with a unique node.

Algorithm 1 delineated how the GN model performs inbound packet classification using just the two attribute values of *lp* and $\Delta_{t}$ to accurately predict each node type in the live IoT dataset (Section 4). To ensure an equitable evaluation, all ML comparators were similarly trained on the same two attributes and their related values also directly extracted from the live IoT dataset.

Using the GN model as a benchmark for detection performance, the corresponding evaluation metrics in Table 7 conclusively demonstrate its consistently superior accuracy to all ML classifiers, along with an analogous *False Positive Rate* (FPR) to J48.

Table 7 shows that the GN Slow DoS detection model provides a detection accuracy (Equation (7)) of 98.75% which is significantly higher than the ML comparators for TP3, and while this high detection accuracy is impressive, another insightful performance evaluation metric of Slow DoS detection methods is minimising the number of *false positives*, i.e., where an MN erroneously gains network access due to being wrongly classed as an LN or SN. This is measured by the *Precision*, which is defined in Equation (8). The correspondingly high precision value of 0.981 for the GN in Table 7 cogently reflects the low likelihood of *false positive* detections arising, which, coupled with the high *True Positive Rate* (TPR) (or *Recall*) value, collectively validates the superior detection performance of the GN Slow DoS model compared to its resource-intensive ML comparators.

(7) $$\begin{array}{c} \mathrm{Accuracy}=\frac{\mathrm{True}\;\mathrm{Positives}+\mathrm{True}\;\mathrm{Negatives}}{\mathrm{Total}\;\mathrm{Number}\;\mathrm{of}\;\mathrm{Cases}} \end{array}$$

(8) $$\begin{array}{c} \mathrm{Precision}=\frac{\mathrm{True}\;\mathrm{Positives}}{\mathrm{True}\;\mathrm{Positives}+\mathrm{False}\;\mathrm{Positives}} \end{array}$$

ML approaches to intrusion detection can yield high accuracy and precision values when trained and tested on high-volume datasets, involving multiple attributes [21]; however, it is pragmatically argued that they are less effective when the detection model uses only two attributes, and the traffic profile includes high-latency SNs. To better contextualise potential SN misclassifications, Table 8 displays the comparative confusion matrix results for the GN and J48 classifier (in parenthesis), where the latter has 42 instances of SN packets, (≈9%) incorrectly classified as an MN for TP3.

In contrast, the GN model has a significantly lower number of SN misclassifications, thus demonstrating its enhanced capability to differentiate genuine SNs from MN traffic. The results also reveal that the *Precision* mean value across the three classes is 0.985, which is again higher than the best ML classifier J48.

#### Evaluating Time Constraints

Furthermore, as inculcated in Section 2, a drawback of ML models is the sizeable investment required in computational time and network resources for continual testing and retraining [1]. This is confirmed in Table 7, where the right-hand column displays the time incurred in running the six ML classifiers compared to the GN detection model, which processes incoming packets in real time.

Recently, [44] utilised Bayesian and Random Forest classifiers to detect DoS in IoT environments, using 29 attributes which were extracted from the Bot-IoT dataset. The model’s computational times cited for training and testing range from 6 to 14 min, and 15 to 31 s, respectively, further ratifying the performance benefits of the GN model’s lightweight two-attribute detection methodology.

### 6.2. Resource Efficiency

The key advantage of the GN detection model is its high resource efficiency, and while acknowledging (see Section 3) that an extra IoT node (GN) is introduced, in comparison with this inexpensive lightweight device, ML approaches incur computationally intensive, high-specification servers to manage large volumes of data. Using the processing cost relationship in [46], the overhead can be determined from the number of times an HTTP application $\left(c_{a}\right)$ generates processor instructions. This may be approximated by considering the processing time in terms of packets per second ($\beta_{a}$), mean packet length ($lp_{\mu }$), and application running time ($t_{a}$), as formalised in Equation (9). The comparative resourcing results for the GN model, *Full Packet Capture* (FPC) and inbound-only packet capture are displayed in Table 9. This reveals substantial savings across a range of resource metrics by the GN model, with a respective processing saving of 67% compared to FPC used in many ML approaches [1] and state of the art [7], and over 65% to inbound-only packet analysis [11]. In addition, storage savings of up to 96% are achieved compared with the ML solution in [13].

(9) $$C_{a}=\frac{\beta_{a}\times lp_{\mu }}{t_{a}}$$

**Figure 4:**
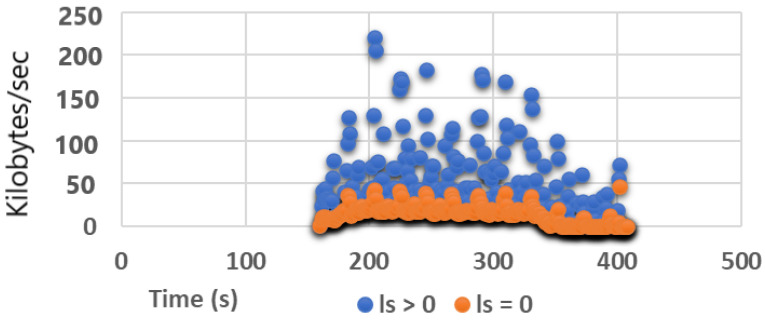
Comparative traffic throughput in kilobytes/sec for the SG node.

To validate the significant cost savings achieved by the GN model in terms of processing efficiency and storage requirements, Figure 4 displays SG attack traffic throughput recorded over a 246 s period. By only inspecting packets where ls=0 bytes, throughput is reduced by 96% compared to FPC, and 62% with all inbound packet capture. Similar reductions are observed for the other two Slow DoS variants SR and SP, yielding 99% and 98% savings, respectively, against FPC, and 59% and 60% for inbound-only packet capture. The important point to inculcate in these results is these resource savings for the GN model are achieved without compromising Slow DoS detection accuracy.

Finally, the new GN model affords one other tangible benefit in being a far more financially attractive Slow DoS detection alternative to ML approaches, which generally incur higher financial costs in their resourcing, hardware, and third-party provider overheads.

## 7. Conclusions

Malicious Slow *DoS* (Denial of Service) attacks on HTTP application layer services are very hard to reliably detect as they masquerade as legitimate network traffic, often resembling nodes with slow connectivity. This paper has presented a novel *Guardian Node* (GN) Slow DoS detection model that uniquely detects the Slow Get, Slow Read, and Slow Post attack variants in a highly resource-efficient manner, and is thus especially germane for resource-scarce IoT environments. The model presented in this paper shows improved detection accuracy compared to various *machine learning* (ML) classifiers when subjected to only two network attributes for detection modelling. In contrast to existing Slow DoS detection solutions, which are primarily based upon computationally intensive ML, and generally involve both full packet capture and post-event processing, the GN model operates in real time and demonstrates comparably high detection accuracies. By analysing only inbound packets that fulfil a prescribed network attribute criterion, allied with fine-tuning of the key attribute parameters of packet length and packet delta time, all three Slow DoS attack variants can be accurately detected, while crucially still distinguishing legitimate nodes from slow IoT nodes encountering poor or intermittent connectivity, thereby ensuring they are not wrongly denied network access. Critical evaluation of the GN model has presented compelling evidence of its detection performance for all Slow DoS variants, even under the stealthiest attack conditions and highly variable traffic loads, with minimal resource expenditure compared with existing ML approaches. This comparison of nodes with slow or poor network connectivity is a critical and unique inclusion in the field of Slow DoS detection and mitigation.

Future work aims to extend the proven accuracy and resource efficiency of the GN detection model by designing an adaptive delta time threshold mechanism. In addition, a formal computational and resource analysis for the GN model will be developed, including comparative delay graphs for detecting Slow DoS attacks.

## Figures and Tables

**Table 1:** Configured connection parameters for each traffic profile.

	TP1	TP2	TP3
Slow Get (cnx/rpc)	2000/200	1000/100	500/50
Slow Read (cnx/rpc)	2000/200	1000/100	500/50
Slow Post (cnx/rpc)	2000/200	1000/100	500/50

**Table 2:** Class frequency for TP1.

Class	Frequency	Percent	Cum. Percent
LN	2601	24.8	24.8
MN	5700	54.3	79.1
SN	2197	20.9	100.0
Total	10,498	100.0	

**Table 3:** Class frequency for TP2.

Class	Frequency	Percent	Cum. Percent
LN	1789	9.5%	9.5
MN	16,078	85.2%	94.7
SN	997	5.3%	100.0
Total	18,864	100.0	

**Table 4:** Class frequency for TP3.

Class	Frequency	Percent	Cum. Percent
LN	1279	26.1%	26.1%
MN	2912	59.4%	85.4%
SN	714	14.6%	100.0%
Total	4905	100.0%	

**Table 5:** Mean delta times ($\Delta t_{\mu }$) for each traffic profile (ms).

Node	TP1	TP2	TP3
Slow Get	4.11	3.74	3.53
Slow Read	4.53	3.21	2.75
Slow Post	3.63	3.43	1.93
Slow Node	1.33	1.25	1.83
Legitimate Node	0.28	0.26	0.27

**Table 6:** Comparative packet length (*lp*) percentages.

	*lp* = 60	*lp* = 66	*lp* = 74	*ls* = 0
SR	6.9	85.3	7.8	92.7
SG	1.6	23.7	74.5	61.9
SP	1.3	38.1	58.2	77.1
SN	99.4	0.6	0.0	80.1
LN	99.4	0.6	0.0	79.4

**Table 7:** Comparative performance results for the GN model and 6 established ML classifiers.

Model	ACC %	TPR (Recall)	FPR	Precision	Time (ms)
Guardian Node	98.75	0.994	0.022	0.985	≤10
BayesNet	91.80	0.976	0.122	0.854	520
REPTree	92.56	0.926	0.053	0.922	710
J48	92.14	0.979	0.022	0.979	660
Random Forest	92.88	0.929	0.050	0.925	53,480
JRip	91.37	0.914	0.067	0.907	22,540
KNN	92.86	0.929	0.051	0.927	337,950

**Table 8:** Comparative confusion matrix results for the GN model and J48 (in parenthesis).

	LN	SN	MN
LN	887 (786)	5 (80)	9 (35)
SN	10 (246)	473 (204)	9 (42)
MN	5 (13)	4 (15)	2011 (1992)

**Table 9:** Benchmark resources comparison (processing and storage) metrics.

Resources Metric	FPC	Inbound	GN
Average bytes per second	168k	14.5k	5.3k
Packets processed (KB)	11,756	7286	2897
Average packets per second	275.2	102.4	79.9
Average packet size	612	55.8	126
CPU load (%)	27.6	18.6	11.9
CPU peak load (Watts)	19.6	17.9	17.1
Storage (MB)	589	44.3	21.5
Processing Cost ($C_{a}$)	50.7	48.7	16.9

## Data Availability

The dataset utilised in this study is available at https://ordo.open.ac.uk/articles/dataset/HTTP_DoS_Dataset_in_PCAP_format_for_Wireshark/17206289 (accessed on 1 June 2024).

## Abbreviations

The following abbreviations are used in this manuscript:

GN	Guardian Node
SN	Slow Node
SG	Slow Get
SR	Slow Read
SP	Slow Post
LN	Legitimate Node
MN	Malicious Node
TP	Traffic Profile
lp	TCP/IP Packet Length
$\Delta_{t}$	Packet Delta Time
